# Social Networks, Engagement and Resilience in University Students

**DOI:** 10.3390/ijerph14121488

**Published:** 2017-12-01

**Authors:** Elena Fernández-Martínez, Elena Andina-Díaz, Rosario Fernández-Peña, Rosa García-López, Iván Fulgueiras-Carril, Cristina Liébana-Presa

**Affiliations:** 1 SALBIS Research Group, Faculty of Health Sciences, University of León (Spain), 24071 León, Spain; elena.fernandez@unileon.es (E.F.-M.); rgarcl@unileon.es (R.G.-L.); cliep@unileon.es (C.L.-P.); 2 SALBIS Research Group, Department of Nursing, University of Cantabria (Spain), 39008 Santander, Spain; roser.fernandez@unican.es (R.F.-P.); ivafulcar95@gmail.com (I.F.-C.)

**Keywords:** social network analysis, resilience, engagement, students nursing

## Abstract

Analysis of social networks may be a useful tool for understanding the relationship between resilience and engagement, and this could be applied to educational methodologies, not only to improve academic performance, but also to create emotionally sustainable networks. This descriptive study was carried out on 134 university students. We collected the network structural variables, degree of resilience (CD-RISC 10), and engagement (UWES-S). The computer programs used were excel, UCINET for network analysis, and SPSS for statistical analysis. The analysis revealed results of means of 28.61 for resilience, 2.98 for absorption, 4.82 for dedication, and 3.13 for vigour. The students had two preferred places for sharing information: the classroom and WhatsApp. The greater the value for engagement, the greater the degree of centrality in the friendship network among students who are beginning their university studies. This relationship becomes reversed as the students move to later academic years. In terms of resilience, the highest values correspond to greater centrality in the friendship networks. The variables of engagement and resilience influenced the university students’ support networks.

## 1. Introduction

Resilience is an individual’s capacity to respond to stress in a healthy manner, such that he or she can achieve goals at the lowest physical and psychological cost. In relation to students, it is considered a key skill [1,2] because of its direct relationship with students’ mental health, psychological well-being, commitment, achievement, quality of attention [3], self-efficacy, and creativity [4].

On the other hand, engagement is a work-related, positive or satisfactory, persistent cognitive affective state. It is composed of three basic dimensions: vigour (tenacity, effort), dedication (enthusiasm, inspiration, pride, defiance), and absorption (concentration) [2,5]. It is considered to be the opposite of burnout syndrome, and it exerts an equally positive influence on personal and academic performance [1,2], improving psychological well-being, performance and satisfaction, and promoting positive attitudes [6,7,8,9].

Recently, a variety of investigations have focused on directly linking students’ resilience and engagement [10,11,12,13,14,15], and they conclude that more resilient students exhibit higher scores in engagement, and as a result, in academic achievement.

Individuals’ resilience and engagement depends not only on individual factors, but also on other, community-level and institutional ones [16]. The concept of resilience has been integrated into the ecological and development theory, allowing for a multisystemic vision of the resilient behaviors developed by the individual [17]. Along these lines, bioecological theory [18] considers how human development is influenced by the interactions that take place between the individual and the environment. Resilience, although it requires a response of its own, is conditioned by these individual and ambient factors that emerge from the ecological conditions in which the individual develops [19]. On the other hand, the study of resilience of social-ecological systems represented as a network, allows to choose which attributes of the social-ecological system are of interest for the study, as well as the study of those attributes within the structure of the network. In this line, different structural properties of the network may be of interest while using just connectivity and centrality we can capture the essential functional implications for the resilience of the structure of a given socioecological network [20]. Therefore, to enhance these aspects, interventions that are aimed at improving both emotional skills and social [21] ones, such as cultural membership or participation in the community [22], have been suggested.

Following this line of argument, methodology focused on social network analysis (SNA) could become a useful tool in the understanding the relationship between resilience and engagement by focusing attention on social variables of a networked or relational nature.

Social network analysis is a distinctive research perspective within the social and behavioural sciences that encompasses a set of methods, models, and applications that are expressed in terms of relational processes or concepts. It is based on the assumption that relationships between interacting units are important [23]. Two elements make up a social network: a finite set of actors called nodes, which are defined by their attributes or characteristics, and the links that bind these actors and that are defined by their relational characteristics or properties—for example, exchanges of information, friendship, influence, or other relational elements. Unlike conventional social research that is based on actors and attributes, the relevance of SNA lies in the way in which the individual is integrated into a structure, in which the actors have or share certain links, and in how that structure and that interaction with others determine behaviour. It thus prioritizes the characteristics of the social environment, in which the individual is immersed as explanatory variables of the phenomenon under study [24,25,26].

Students configure their contacts, and through doing so, they establish ties of friendship, assistance, and information exchange that may have a positive impact on their academic performance. Some of those contacts have their origins not only in the informal relations that are present outside the classroom, but also in educational methodologies themselves. This generation of processes through interrelation and cooperation inside and outside the classroom, which in SNA is described as the creation of networked structures, is in fact encouraged by the new European Higher Education Area, in order to create added value in curricular paths, the exchange of information, increased efficiency in problem solving, comprehension of concepts, and discussion of views [27].

Along these lines, recent research has shown that the position occupied by a student within communication networks is positively correlated with performance [28] and the achievement of his or her objectives [29]. The act of constructing cooperative networks in students’ learning/teaching process can increase their engagement, knowledge transfer, and academic results [30], and it serves as a cushion if they develop burnout syndrome [31].

Although students develop many contacts within their social environment, which are often highly emotionally loaded, there is a gap in the literature on studies that jointly address the characteristics of student social networks, resilience, and engagement. This leads us to pose the following question: What is the relationship between the structural characteristics of student networks and students’ engagement and resilience? This innovative suggestion may make it possible to ascertain what contact patterns are aligned with engagement and resilience and where these processes take place. Such insight may be applied to educational methodologies, not only to improve academic performance, but also to create emotionally sustainable networks.

Accordingly, we will focus on university students, and specifically nursing students. We do so because, in addition to the fact that they face the typical difficulties associated with adapting to the university environment, they also encounter other difficulties that are specific to the profession that they are training for—for example, intimate care for patients and exposure to communicable diseases or death. These can cause a high degree of discomfort or anxiety, and resilience and engagement can be useful when faced with them [32].

The objectives proposed in this research are:

- To quantify the degree of resilience and engagement (absorption, dedication and vigour) of a group of university students according to the academic year that they belong to.
- To graphically represent the sociocentric networks of contact of the three academic years.
- To identify what level of similarity students have when selecting the places where they share information.
- To analyse the relationship between the centrality structural variables of the class’s network of contacts and students’ engagement and students’ resilience.

## 2. Materials and Methods

This is a transversal descriptive study.

### 2.1. Sample Description

The sample comprises the 134 nursing students that are enrolled at a public university in Spain. These individuals participated voluntarily after being informed about the study. As Table 1 shows, 48 students were in the first year, 44 in the second, and 42 in the third.

### 2.2. Variables

Sex and course year were variables that describe the characteristics of the students. In addition, the following variables have been evaluated:

- Composition variables. Sex and course year were the attributes selected for the present study.
- Engagement and its three dimensions of absorption, dedication and vigour.
- Resilience.
- Centrality structural variables: degree, indegree, outdegree, closeness, eigenvector, and betweenness of each of the participants. By calculating the outdegree, it is possible to represent the links that go from the node to the components of his or her class. Computing the indegree reveals relationships that go from other participants toward the node. We also considered it appropriate to determine degree, which takes both situations into account. In addition, we decided to stipulate the centrality of proximity (closeness), the centrality of intermediation (betweenness) and the degree of influence of each actor (eigenvector) [33].

### 2.3. Data Collection Instruments

Data collection was carried out during the first semester of the academic year 2016–2017, through a self-completed questionnaire with an ad hoc design. The questionnaire collected the following information:

- Students’ sex and course year.
- The UWES-S scale to measure student academic engagement. This questionnaire consists of 17 items that are grouped along three dimensions (absorption, dedication, and vigour), and it evaluates the level of agreement or disagreement on each of them with a range that goes from 0 to 6 [5,34].
- The Connor-Davidson resilience scale, specifically the abridged version by Campbell-Sills and Stein [35], which has been validated for young Spaniards by Notario et al. [36] in 2011: the CD-RISC 10. This instrument, with 10 items, evaluates the level of agreement or disagreement in relation to each of them with a range that goes from 0 to 4.
- Variables for the structure of the students’ network of contacts. To calculate the centrality of Type 1 Networks (Support and Friendship Networks), we used a limited actor census and a 0 to 4 Likert scale with two items (item 1: to whom you ask for help and item 2: who is your friend), to determine the intensity of the relationship. For Type 2 Networks (where to share information), we offered a list of places (class, library, campus corridors, campus cafe, other cafes, gym, email, and WhatsApp), as well as the possibility of adding options where the respondents felt it appropriate to do so. A Likert scale (from 0 to 4) and two items (item 1: where the academic information is shared and item 2: where they share personal information) were used to measure frequency.

### 2.4. Procedure

The first step that was required to analyse the centrality structural variables for sociocentric networks of students in class was to build matrices of each course separately, which describe the relationships of friendship and support that are established in each of the three year groups of the nursing degree. Data was transferred to square matrices of N rows by N columns. Given that the data were collected using a 0 to 4 Likert scale, the relationships between each pair of students were described in values ranging between those numbers. To calculate centrality, it was necessary to introduce dichotomized data (Table 2).

For the Support Network, the following dichotomization criterion was taken. Answers ranging between 0 and 1 (“never” or “rarely”) would represent a 0, which corresponds to “does not ask for help”, while values between 2 and 4 (“sometimes”, “often” and “always”, respectively) were represented with a 1, which corresponds to “asks for help” [37].

In the case of Friendship Network, dichotomization was produced according to three criteria to reflect different friendship intensities. In the first dichotomization, 0 (nothing) was considered “no friendship” and 1–4 (“not much friendship”, “somewhat friends”, “quite good friends” and “very good friends”) were represented with a 1, which corresponds to “presence of friendship”. The resulting network was named “minimum friendship network”. In the following dichotomization the value 1 (“not much friendship”) is represented as a 0, which corresponds to “no friendship”, and 2–4 (“somewhat friends”, “quite good friends” and “very good friends”) were represented as 1, “presence of friendship”, meaning that the friendship relationship represented here is more intense than the previous one. We decided to name this network the “Intermediate friendship network”. The last matrix was dichotomized by considering 0–2 as “no friendship” and only the values 3 and 4 (“quite good friends” and “very good friends”) as 1, “presence of friendship”, thereby once again increasing the intensity of the link between actors. This last network was called the “maximum friendship network” [38].

To analyse Type 2 or affiliation networks, where the matrices contain N rows of actors and nine columns of places or events, the data were also dichotomized. In this case, and for both matrices per course, it was decided that the responses 0 (never), 1 (1 or 2 times per week) and 2 (3 or 4 times per week) were interpreted as places, events or communication channels where information is not regularly shared, while responses 3 and 4 (5 or 6 times a week and daily) were considered to be places where information is regularly shared. For this analysis, the responses of the students from the three years were transferred to a single network, since it was considered that the answers would be more similar and because we were interested in an overview of the entire network.

### 2.5. Data Analysis

The data obtained were transferred to Excel and processed using the program UCINET (V.6.365, Analytic Technologies, Lexington, KY, USA) [39], through which we calculated the measures of centrality (degree, indegree, outdegree, closeness, eigenvector, and betweenness) of each of the subjects. All these data were normalized, and we created graphs of the type 1 and type 2 networks. According to the graphic representation of the networks, the position nodes are always analysed with the algorithms applied for UCINET [40].

Once we had obtained the centrality data through UCINET, the results were exported to the program SPSS (V.24, IBM, New York, NY, USA) for descriptive analysis, as well as for the correlations between the variables of centrality, engagement, and resilience.

### 2.6. Ethical Considerations

The surveys were accompanied by an information sheet and verbal and written consent. At all times we considered the anonymity of the subjects who were part of the study, and so the names of the actors shown in the graphs are different and fictitious. We also ensured that participation was voluntary.

During the study, we followed national and international guidelines (Code of Ethics and Declaration of Helsinki), and we followed the legal regulations on data confidentiality (Spain’s organic law 15/1999 of 13 December on the protection of personal data). The study was approved by the university’s ethics committee (ETICA-ULE-010-2017), thereby ensuring that there was compliance regarding ethical and legal matters.

## 3. Results

The results show (see Table 3) that relative to the variable engagement, the dimension of dedication attained the highest value with an average of 4.82. The mean for resilience was 28.61.

**Figure 1:**
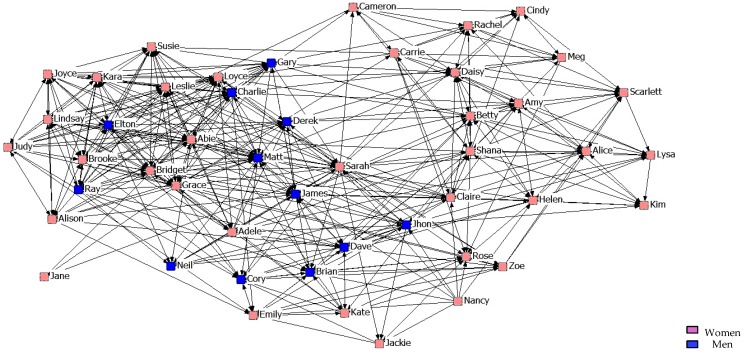
Graph of the first year Support Network.

**Figure 2:**
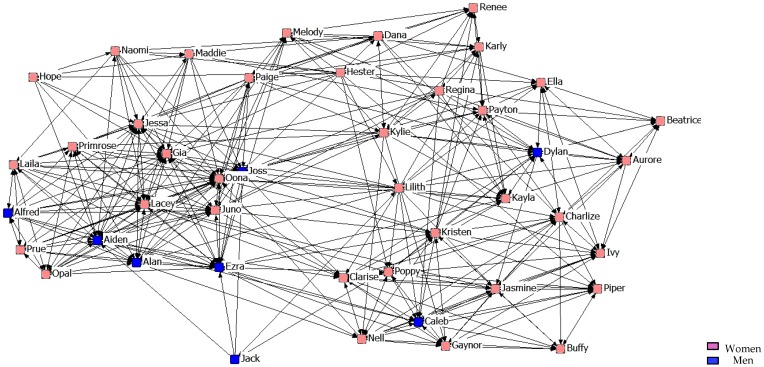
Graph of the second year Support Network.

**Figure 3:**
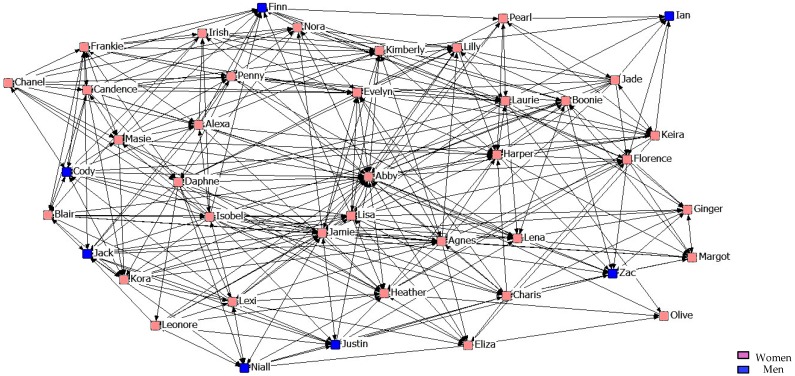
Graph of the third year Support Network.

With regard to the results of the graphical representation of the networks, Figure 1, Figure 2 and Figure 3 represent networks of support for the first, second, and third years. It can be seen that the third-year network has a more uniform arrangement of links, in which male students are in a more peripheral position.

**Figure 4:**
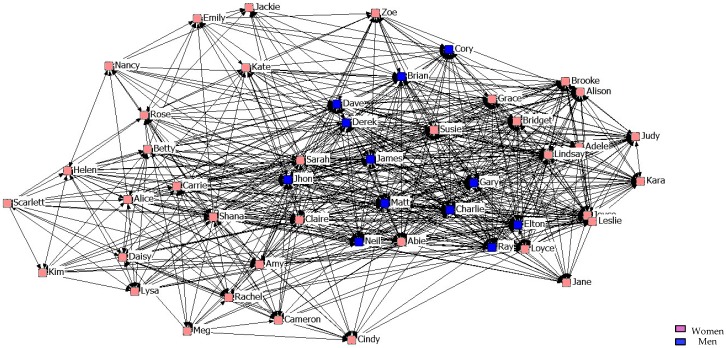
Graph of the first year Minimum Friendship Network.

**Figure 5:**
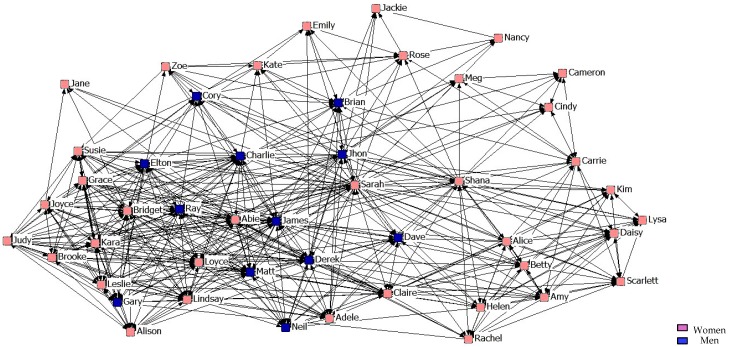
Graph of the first year Intermediate Friendship Network.

**Figure 6:**
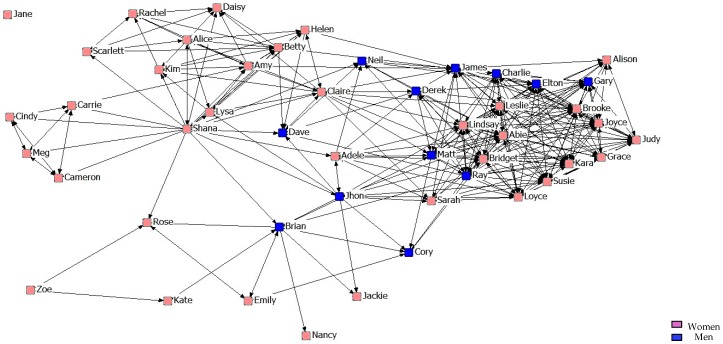
Graph of the first year Maximum Friendship Network.

Figure 4, Figure 5 and Figure 6 describe friendship in the first year. According to the graphs, students perfectly identified a strong friendship, with there being no difference between the sexes.

**Figure 7:**
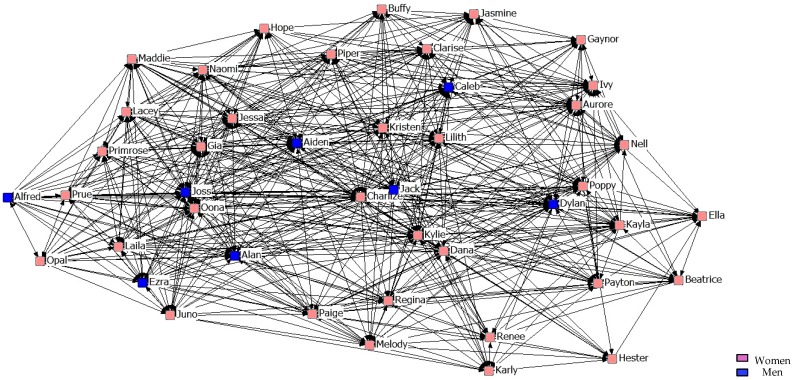
Graph of the second year Minimum Friendship Network.

**Figure 8:**
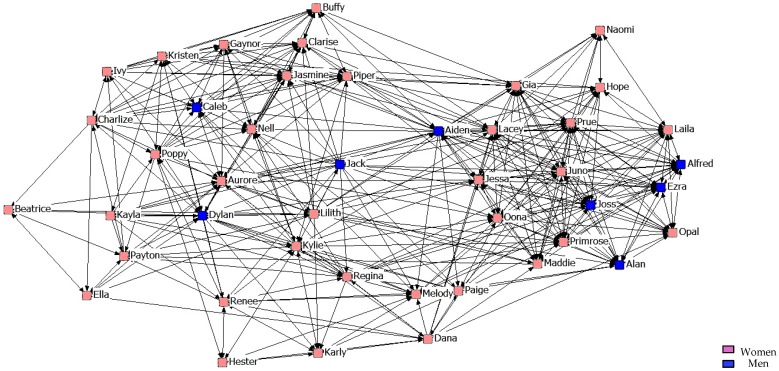
Graph of the second year Intermediate Friendship Network.

**Figure 9:**
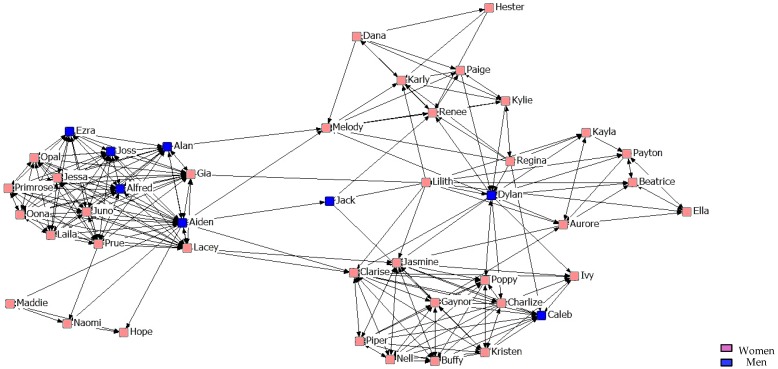
Graph of the second year Maximum Friendship Network.

Figure 7, Figure 8 and Figure 9 describe friendship in the second year. What stands out is that when stronger friendship relations are established, they are more restrictive than those of the students from the previous year.

**Figure 10:**
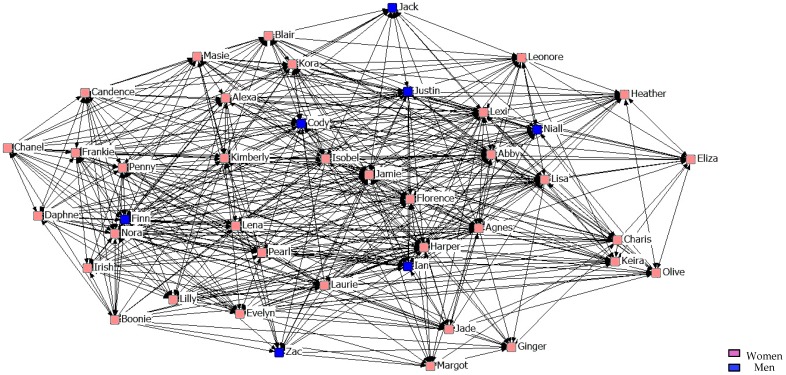
Graph of the third year Minimum Friendship Network.

**Figure 11:**
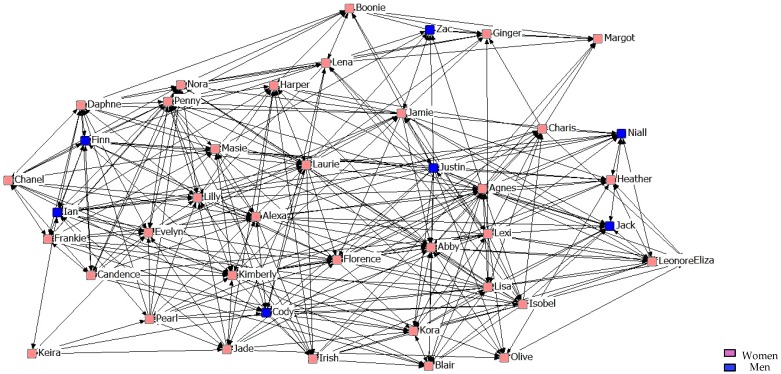
Graph of the third year Intermediate Friendship Network.

**Figure 12:**
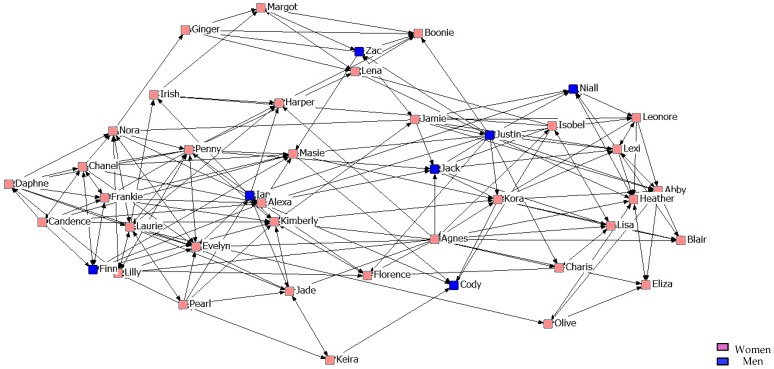
Graph of the third year Maximum Friendship Network.

Figure 10, Figure 11 and Figure 12 describe friendship among students in the third year. We might point out that within the Maximum Friendship Network, the structural subgroups are not so obvious, unlike the case of the two previous years.

**Figure 13:**
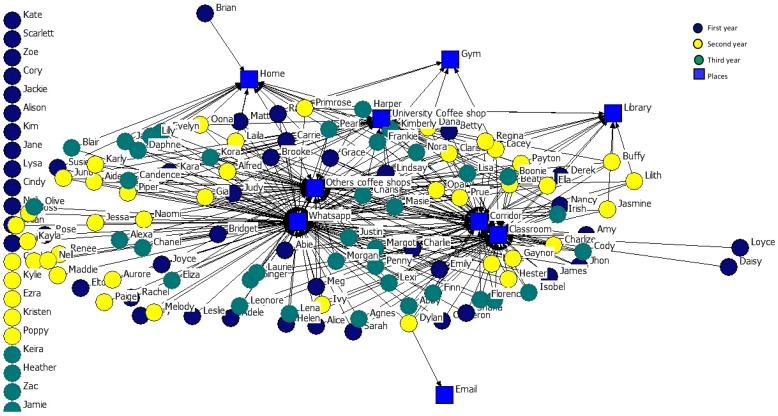
2-mode Network of places where personal information is shared.

**Figure 14:**
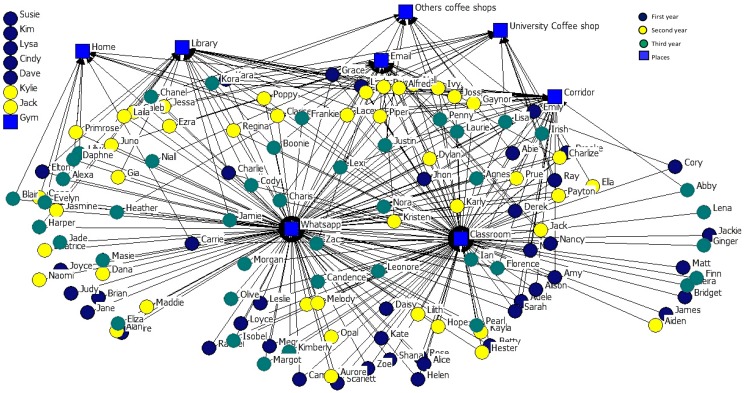
2-mode Network of places where academic information is shared.

In considering academic year as an attribute, in order to represent the places where students share information we prepared two graphs: one for the sociocentric network of the three academic years and the places where they share personal information (Figure 13), and one for the sociocentric network of the three academic years and the places where they share academic information (Figure 14).

It can be seen that students from the first (green nodes) and second (yellow nodes) years, as well as students from the third year (brown nodes), choose similar places to share this type of information (WhatsApp and classroom), and the gym, email communication channel, the library, and the home are spaces in which very few students choose to exchange either type of information. Email is the least used channel to share personal information. Isolated nodes only use a channel to share information.

We analysed correlations between scores for the dimensions of the variable engagement and centrality structural variables in each of the networks obtained. We show the statistically significant correlations (Table 4).

With regard to resilience, in Table 5 we show the statistically significant correlations for the centrality structural variables.

## 4. Discussion

First, and with respect to engagement, students in the first year exhibited higher levels in its three dimensions. This indicates that at the start of their degree, students present greater vigour, are more absorbed in it, and have a more intense level of dedication to academic tasks. This is pointed out in the study by Liébana [41], with findings of 3.21 for absorption, 4.40 for dedication, and 3.08 for vigour. In both works, dedication was the factor in which students obtained higher scores.

In terms of resilience, first-year students presented a higher mean relative to the other two years. If we compare the mean for resilience that was obtained by the entire sample with the study by Ríos et al. [42], which analysed this variable in students studying the same degree, we find that the students in our study obtained a lower mean, since the mean in that study was 34.70.

It was not possible to establish a comparison of the results obtained according to the year of study with other works, because our search of the literature did not bring up any related studies. On the other hand, we found that after students move to later years, the results remained concordant, raising the possibility that differences were found due to the student’s year or that these are traits that were peculiar to the individuals in the sample.

The places for sharing both academic and personal information that were most frequently cited by students were class and WhatsApp, regardless of the year of study. Durling, Tomas, and Grunspan indicate in their research that the classroom is where such exchanges take place [27,43,44]. Young people use digital social networks as a way to share all kinds of information [45,46], and WhatsApp is the messaging application that is most widely used by young people because of convenience, lack of time, or problems with shyness [47], and so its inclusion is not surprising.

With the data obtained in the correlations between the centrality structural variables and engagement, students in the first year who presented a greater degree of absorption (i.e., those who may be most enthusiastic about their degree), also had higher centrality in the friendship network (more friends and more prestige among them). We can thus point out that students positively assessed others’ engagement when establishing a friendship with them, though we must do so with some caution, because, as this study was descriptive and transversal, we cannot be fully certain what the cause was and what the consequence was. In the second and third years the opposite happened, and the students who focused more on academic subjects (with higher values in vigour and absorption) had fewer friends. That is, higher engagement figures supposed a difficulty in establishing or maintaining friendships. This is understandable if we consider that, as the degree’s course units become complicated and friendships have already been forged, students do not seek to expand their relationships with their peers. In the literature, we equally found in this respect direct associations between values of high engagement and scarce free time [48], as well as high levels of engagement and high motivation to achieve good academic results [30].

Finally, by correlating centrality structural variables and resilience, we found that the highest values for resilience were obtained from the most central students in the network, regardless of the academic year that they were in. Moreover, we established a positive correlation between students who presented the highest values of resilience and those who had a greater degree of relations, not only through being the most named, but also through naming more of their peers. In addition, these individuals that were considered to be more resilient had greater influence and intermediation within the network. Therefore, one would expect that the friendship network might be favoured if an individual was more resilient. This association between resilience and student support systems has been similarly pointed out in different studies [1,49,50], and so it would be interesting to delve into these support systems with network studies, since from a conceptual perspective, support systems are similar to support networks.

This study presents some limitations: the transversal and descriptive nature and voluntary participation. In addition, only webbased social networks (email and WhatsApp) have been taken into account for the exchange of personal or academic information. The conclusions cannot be generalized to other academic fields since all of the subjects are nursing students.

The results that were obtained can guide us in terms of how networks of relationships between university students are formed, how they are distributed, how they evolve, and what possible relations they may have with engagement and resilience. In this sense, and in the words of Friemel: “The context consists of all information which would be of interest to a research question less the information represented by the units of recording. Consequently, the question is not if context matters (because it matters by definition) but rather why context matters”. In this way, this line of research or studies labeled “explanative applications” of Social Network Analysis, try to explain how attributes of individual units are dependent on their structural embedding within a set of other units and their respective attributes, underlining in this way again, the importance of the social and relational context [51].

As future lines of research, it would be desirable to perform longitudinal studies in students from different degrees, in which, through methodologies based on SNA, our knowledge of the role played by the structural features of networks of students in relation to resilience and engagement would be improved. It would be interesting to use the Principal Component Analysis (PCA) in order to learn about the relationships between the structural characteristics and the social networks of the students and the engagement and resilience. When considering the modern impact of webbased social networks, more attention should be directed to this issue when figuring possible studies in this domain.

## 5. Conclusions

We have managed to fulfil the objectives outlined in this study. We quantified the degree of resilience and engagement (absorption, dedication and vigour) of a group of university students according to their year of study, and found higher values at the beginning of students’ university education.

Moreover, we graphically represented the sociocentric contact networks of the three academic years, identifying what degree of similarity students had when choosing the places where they share information.

In addition, we analysed relations between the centrality structural variables, engagement, and resilience, concluding that the greater the degree of engagement, the greater the degree of centrality in the friendship network among students who are beginning their university studies. This relationship becomes reversed as the students move to later academic years. In terms of resilience, the highest values correspond to greater centrality in the friendship networks.

As a practical application, academic leaders should implement programs to promote engagement and resilience in university students in order to improve the communication network.

## Figures and Tables

**Table 1:** Student characteristics.

Study Year	Sex		Total *N* (%)
	Men *N* (%)	Women *N* (%)	
First year	12 (25%)	36 (75%)	48 (100%)
Second year	8 (18%)	36 (82%)	44 (100%)
Third year	7 (17%)	35 (83%)	42 (100%)
Total	27	107	134 (100%)

**Table 2:** Dichotomization process, Type 1 Support and Friendship Networks.

Dichotomization: Support Network		First Dichotomization: Minimum Friendship Network		Second Dichotomization: Intermediate Friendship Network		Third Dichotomization: Maximum Friendship Network	
No presence of support	Presence of support	No presence of friendship	Presence of friendship	No presence of friendship	Presence of friendship	No presence of friendship	Presence of friendship
0, 1	2, 3, 4	0	1, 2, 3, 4	0, 1	2, 3, 4	0, 1, 2	3, 4

**Table 3:** Descriptive results of students’ engagement and resilience.

Variables		*N*	Mean	Standard Deviation
Engagement—Absorption	First year	48	3.2748	1.03296
	Second year	44	2.8018	1.02480
	Third year	42	2.8362	0.99771
	Total	134	2.9820	1.03530
Engagement—Dedication	First year	48	4.8333	0.92560
	Second year	44	4.7545	0.76537
	Third year	42	4.8810	0.70684
	Total	134	4.8224	0.80549
Engagement—Vigour	First year	48	3.3956	1.08313
	Second year	44	2.9170	1.16347
	Third year	42	3.0571	1.01136
	Total	134	3.1324	1.09985
Resilience	First year	48	29.42	5.181
	Second year	44	27.57	6.241
	Third year	42	28.79	5.092
	Total	134	28.61	5.539

**Table 4:** Correlations between engagement and centrality variables.

Network	Engagement	Centrality V.	Pearson C.
First year. Minimum Friendship Network	Absorption	Outdegree	0.351 *
		Degree	0.308 *
		Closeness	0.332 *
		Betweenness	0.403 **
First year. Intermediate Friendship Network		Outdegree	0.318 *
		Degree	0.286 *
		Closeness	0.291 *
		Betweenness	0.326 *
First year. Maximum Friendship Network		Betweenness	0.308 *
First year. Minimum Friendship Network	Vigour	Betweenness	0.293 *
Second year. Intermediate Friendship Network	Absorption	Eigenvector	−0.346 *
	Vigour	Eigenvector	−0.303 *
Second year. Maximum Friendship Network	Absorption	Eigenvector	−0.389 **
	Dedication	Outdegree	0.303 *
	Vigour	Eigenvector	−0.350 *
Second year. Support Network	Dedication	Degree	0.315 *
	Absorption	Eigenvector	−0.299 *
Third year. Minimum Friendship Network	Vigour	Indegree	−0.317 *
Third year. Intermediate Friendship Network	Absorption	Indegree	−0.331 *

* Correlation is significant at the 0.05 level. ** Correlation is significant at the 0.01 level.

**Table 5:** Correlations between resilience and centrality variables.

Network	Centrality V.	Pearson C.
First year. Minimum Friendship Network	Indegree	0.322 *
First year. Intermediate Friendship Network		0.291 *
Third year. Minimum Friendship Network	Outdegree	0.346 *
	Degree	0.324 *
	Eigenvector	0.350 *
Third year. Intermediate Friendship Network	Outdegree	0.400 **
	Degree	0.330 *
	Closeness	0.342 *
	Eigenvector	0.325 *
Third year. Maximum Friendship Network	Outdegree	0.331 *
	Degree	0.369 *
	Closeness	0.309 *
	Eigenvector	0.366 *
	Betweenness	0.334 *
Third year. Support Network	Outdegree	0.312 *

* Correlation is significant at the 0.05 level. ** Correlation is significant at the 0.01 level.
